# Spatial‐Temporal Assessment of Environmental Factors Related to Dengue Outbreaks in São Paulo, Brazil

**DOI:** 10.1029/2019GH000186

**Published:** 2019-08-21

**Authors:** I. Ogashawara, L. Li, M. J. Moreno‐Madriñán

**Affiliations:** ^1^ Department of Earth Sciences Indiana University‐Purdue University at Indianapolis Indianapolis IN USA; ^2^ Department of Environmental Health, Fairbanks School of Public Health Indiana University‐Purdue University at Indianapolis Indianapolis IN USA

**Keywords:** dengue, El Niño, remote sensing indices, land surface temperature, Landsat 8, continuous wavelet transformation

## Abstract

Dengue fever, a disease caused by a vector‐borne flavivirus, is endemic to tropical countries, but its occurrence has been reported worldwide. This study aimed to understand important factors contributing to the spatial and temporal patterns of dengue occurrence in São Paulo, the largest municipality of Brazil. The temporal assessment of dengue occurrence covered the 2011–2016 time period and was based on climatological data, such as the El Niño indices and time series statistical tools such as the continuous wavelet transformation. The spatial assessment used Landsat 8 data for years 2014–2016 to estimate land surface temperature and normalized indices for vegetation, urban areas, and leaf water. Results from a cross correlation for the temporal analysis found a relationship between the sea surface temperature anomalies index and the number of reported dengue cases in São Paulo (*r* = 0.5) with a lag of +29 (weeks) between the climatic event and the response on the dengue incidence. This relationship, initially nonlinear, became linear after correcting for the lag period. For the spatial assessment, the linear stepwise regression model detected a low relationship between dengue incidence and minimum surface temperature (*r* = 0.357) and no relationship with other environmental parameters. The poor relationship might be due to confounding effects of socioeconomic factors as these seem to influence the spatial dynamics of dengue incidence. More testing is needed to validate these methods in other locations. Nevertheless, we presented possible tools to be used for the improvement of dengue control programs.

## Key Points

- The temporal analysis found a relationship between the sea surface temperature anomalies (SSTA) index and dengue incidence in São Paulo
- The spatial assessment detected a low relationship between dengue incidence and temperature but not with other environmental parameters
- The low relationship between the physical environment and dengue incidence may be explained by confounding effects of socioeconomic factors

## 1 Introduction

Approximately 390 million dengue infections occur globally every year (Bhatt et al., 2013). Dengue outbreaks have been reported in many countries in Asia and South America such as Brazil, Cambodia, Ecuador, Peru, Philippines, Singapore, and Thailand (Halsted, 2007), and it has been also reported in North America (CDC, 2010). In Brazil, dengue outbreaks have been reported since the 1980s, especially during the rainy season. However, in the last decade, the number of cases drastically increased (Aguiar et al., 2015).

Dengue is an acute febrile disease caused by a virus (Phillips, 2008), and it has four strains (Halsted, 2007). The most common carriers are *Aedes* mosquitoes, especially the *Aedes aegypti*. These mosquitoes are usually found in tropical urban areas, especially in places where there is rain water accumulation (Focks et al., 1995). In addition to rainwater, climatic characteristics such as air temperature, humidity, and solar radiation encourage *Aedes* mosquito to grow (Patz et al., 2005). Because of this, the interannual variability of climate correlates to dengue cases in tropical countries. El Niño indices like the El Niño–Southern Oscillation (ENSO), the Southern Oscillation Index (SOI), and the sea surface temperature anomalies (SSTA) also predict dengue outbreaks (Cazales et al., 2005; Gagnon et al., 2001; Fan et al., 2014; Fuller et al., 2009)

Other indicators of the spread, establishment, and persistence of vector‐borne diseases such as dengue are environmental (Messina et al., 2012). Phillips (2008) showed that the expansion of urban areas is a key driver for the increased endemicity of dengue. Since the urban population is increasing worldwide, dengue cases will grow as the trend continues (Messina et al., 2012). Other environmental factors that predict dengue include terrain elevation and vegetation (Fuller et al., 2009; Moreno‐Madriñán et al., 2014; Stanford et al., 2016).

The variety of factors that lead to rises in dengue cases calls for the development of tools that predict dengue based on its geographical distribution in the past (Messina et al., 2012). For other environmental applications, the combinations among the spatial distribution of environment parameters are used to perform risk assessments through statistical or mechanical modeling of environmental trends. A growing alternative to monitor environmental trends, especially in resource‐limited regions, is the use of remote sensing. Hadjimitsis and Clayton (2009) used remote sensing products as predictors for the computation of such models, exploiting advantages such as (1) the synoptic view of satellite or airborne images, which allows the acquisition of data from the entire environment; (2) information from remote and sometimes inaccessible regions; and (3) historical data.

One of the satellite‐derived environmental variables that dengue outbreaks modeling uses most is rainfall, usually from the Tropical Rainfall Measuring Mission satellite (Ashby et al., 2017; Moreno‐Madriñán et al., 2014; Pinto et al., 2011). However, Stanforth et al. (2016) showed that while temperature, elevation, and vegetation cover significantly correlated with dengue outbreaks reported in Magdalena River watershed in Colombia, rainfall did not. Satellite‐derived products such as land surface temperature (LST), digital elevation models, and vegetation indices can be used to compute temperature, elevation, and vegetation cover. However, since the Moderate Resolution Imaging Spectrometer products—usually used for dengue research (Ashby et al., 2017; Buczak et al., 2012; Stanforth et al., 2016)—do not model these factors spatially, they do not model the spatial distribution of dengue cases accurately in the Magdalene River watershed (Stanforth et al., 2016). This calls for a remote sensing approach that can provide a better spatial understanding of the environmental variables.

The goal of this research was to identify the temporal and spatial characteristics of environmental factors affecting the occurrence of dengue in a large urban area, São Paulo, the largest municipality in Brazil. To do this, we evaluated environmental and dengue cases data from the city to determine the temporal and spatial relationships among dengue cases and environmental factors. The evaluations were conducted based on a time series analysis of the data set as well as remote sensing‐derived indices to measure the geographic distribution of environmental factors.

## 2 Material and Methods

### 2.1 Study Site

**Figure 1:**
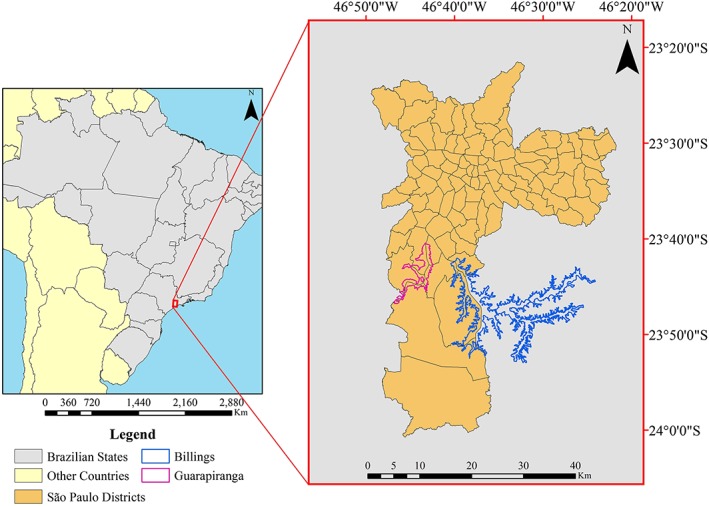
Location of São Paulo Municipality within Brazil and its 96 districts.

At approximately 11 million inhabitants, São Paulo is one of the most populous cities in the world (Prefeitura do Município de São Paulo, 2017). This population is distributed within 1,509 km^2^ of area which consists of 32 subprefectures or 96 districts, as presented in Figure 1. São Paulo is Brazil's major financial and economic center, contributing 11.37% of total Brazilian gross domestic product (IBGE, 2012). However, São Paulo has broad economic diversity, with 13.30% of households living on less than two minimum wages (MWs) jobs, 24.39% living on wages amounting to 2–5 MWs, 25.97% on 5–10 MWs, 11.29% on 10–15 MWs, 10.98% on 15–25 MWs, and 14.06% of families living on more than 25 MWs (SEADE, 2000). The use of MWs as an economic diversity indicator is a distinctive feature of the Brazilian economy. This status is based on the fact that MW affects employment through wages, pensions, benefits, inflation, and the public deficit (Lemos, 2004). Moreover, the Brazilian Institute of Geography and Statistic (IBGE in Portuguese) uses MWs to define the social class of the Brazilian population. Therefore, families living on low MWs represent a lower social class, while a higher MWs represents richer families. This condition along with the urban landscape diversity, the climatic context, and the size of the exposed population make São Paulo a good study site for the current research.

### 2.2 Dengue Data

Confirmed dengue cases for the municipality of São Paulo were acquired on a monthly basis data set from the State Secretariat of Health for the period between 2011 and August of 2017 (São Paulo State Secretariat of Health, 2017). For each district of São Paulo municipality, only data of reported dengue cases were collected from the City Secretariat of Health for the period between 2011 and August of 2017 (São Paulo Secretariat of Health, 2017).

### 2.3 Climatological Data

**Figure 2:**
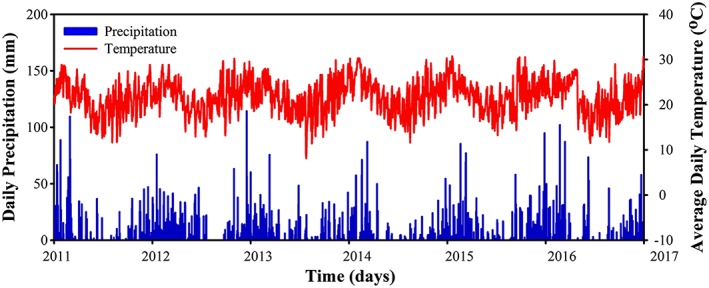
Daily time series (2011–2016) of precipitation (mm) and temperature (°C) from the weather station located at “Mirante do Santana,” São Paulo, Brazil.

Daily temperature and precipitation data from the weather station of Mirante do Santana (23°30′S,46°37′W) were acquired from the National Institute of Meteorology (INMET in Portuguese, 2017) via the Historical Research and Teaching Database. Figure 2 summarizes the data set for the same period of dengue data. It was observed that higher temperature and higher precipitation occurs in the beginning of each year, which is related to the austral summer. Therefore, São Paulo's climate can be classified as humid, subtropical, and oceanic, without a dry season and with hot summers (classified as Cfa based on Köppen's classification method; Alvares et al., 2014).

The most common El Niño indices are the air pressure indices, sea surface temperature indices, and longwave radiation indices. Since sea surface temperature data were recognized to be a key player in ENSO (Rasmussen & Carpenter, 1982), the use of this type of index is more common in the scientific literature. To calculate the sea surface temperature, certain regions were defined for in situ measurements and were divided into four areas: Niño1, Niño2 (combined into Niño1 + 2), Niño3, and Niño4. However, it was identified that a combination between areas 3 and 4 would be the most ENSO‐representative (Barnston et al., 1997). Huang et al. (2013) showed that the area‐averaged Niño‐3.4 SSTA index in the tropical Pacific is more consistent with in situ observations in extended reconstructed sea surface temperature, version 3b. Thus, in this study we decided to use the Niño 3.4 SSTA index. The weekly SSTA index data for Niño region 3.4 (the area within 5°N, 5°S, 120°W) were taken from the Climate Prediction Center of the U.S. National Weather Service (2017). The weekly data set was chosen to match the epidemiological weeks.

### 2.4 Satellite Data

#### 2.4.1 Earth Observation Images

Landsat 8 images from the Operational Land Imager (OLI) and the Thermal Infrared Sensor (TIRS) sensors were used to remotely estimate environmental variables to complete the spatial analysis of this study. TIRS images were acquired around 13:00 UTC time (10:00 in São Paulo) and were used at level 1 processing products to compute LST, while OLI atmospherically corrected products—Landsat 8 Surface Reflectance (L8SR)—were used for the computation of environmental indices. Both products were downloaded from the U.S. Geological Survey (USGS) Earth Explorer website, and Table 1 presents the list of images used.

**Table 1:** Image Identifier, WRS‐2 Path and Row, and Date for the OLI Images Used in This Study

Image identifier	Path/row	Acquisition date
LC82190762014039LGN00	219/76	2014‐02‐08
LC82190772014039LGN00	219/77	2014‐02‐08
LC82190762015010LGN00	219/76	2015‐01‐10
LC82190772015010LGN00	219/77	2015‐01‐10
LC82190762016109LGN00	219/76	2016‐04‐18
LC82190772016109LGN00	219/77	2016‐04‐18

*Note*. Dates are formatted as year‐month‐day. OLI = Operational Land Imager; WRS = Worldwide Reference System.

#### 2.4.2 Environmental Indices

Environmental indices such as the Normalized Difference Vegetation Index (NDVI; Rouse et al., 1973), Normalized Difference Water Index (NDWI; Gao, 1996) and Normalized Difference Built‐up Index (NDBI; Zha et al., 2003) were estimated from OLI‐SR to characterize the environment using remote sensing images. NDVI is one of the most used vegetation indices especially because of its advantages such as lower influence of atmospheric variations, greater sensitivity to chlorophyll, the reduction of noise by normalization between −1 and +1 (Gutman, 1991; Luo & Li, 2014), and the possibility to monitor seasonal changes (Ponzoni & Shimabukuro, 2009). The NDWI is also known as the leaf area water‐absent index and is used primarily to estimate the water content within vegetation (Gao, 1996). However, recent studies have been using NDWI as an index to identify water bodies (McFeeters, 1996; Moknatian et al., 2017; Xu, 2006). Zha et al. (2003) developed the NDBI index to be used to highlight urbanized and barren soil areas.

#### 2.4.3 Estimation of LST

The retrieval of LST from Landsat 8 was based on the measurement from the TIRS. To estimate LST from the TIRS images, we used the single‐channel method, which has been successfully applied in several studies (Jimenez‐Munoz et al., 2009; Jimenez‐Munoz & Sobrino, 2003; Sobrino et al., 2004). To compute LST for Landsat 8/TIRS, we used equation (1), which Jimenez‐Munoz et al. (2014) proposed:

(1) $$\mathrm{LST}=\gamma \left[\varepsilon^{-1}\left(\psi_{1}L_{\mathrm{sen}}+\psi_{2}\right)+\psi_{3}\right]+\delta \;$$

where *LST* is given in Celsius (°C), *ε* is the spectral surface emissivity, *L*_*sen*_ is the top‐of‐the‐atmosphere (TOA) radiance, *γ* is a parameter computed by equation (2), *δ* is a parameter computed by equation (3), and *ψ*_1_, *ψ*_2_, and *ψ*_3_ are derived from the water vapor (*w*) content following the procedure described in Yu et al. (2014, equation 6.

(2) $$\gamma =\frac{T_{\mathrm{sen}}^{2}}{b_{\gamma }L_{\mathrm{sen}}}$$

(3) $$\delta =T_{\mathrm{sen}}-\frac{T_{\mathrm{sen}}^{2}}{b_{\gamma }}$$

where *T*_*sen*_ is the at‐sensor brightness temperature, *b*_*γ*_ is 1324 for Band 10, and 1199 K for Band 11.

*T*_*sen*_ is calculated based on the conversion from digital numbers to TOA radiance (equation (4)), and then from TOA radiance to *T*_*sen*_ (equation (5)) using the thermal constants provided in the metadata file of the Landsat 8/TIRS image (USGS, 2016).

(4) $$L_{\mathrm{sen}}=M_{L}\cdot Q_{\mathrm{cal}}+A_{L}$$

where *L*_*sen*_ is the TOA spectral radiance, *M*_*L*_ represents the band‐specific multiplicative rescaling factor, *Q*_cal_ is the Band 10 image, and *A*_*L*_ is the band‐specific additive rescaling factor.

(5) $$T_{\mathrm{sen}}=\frac{K_{2}}{\ln\left[\left(\frac{K_{1}}{L_{\mathrm{sen}}}\right)+1\right]}-273.15$$

where *K*_1_ and *K*_2_ stand for the band‐specific thermal conversion constants from the metadata.

The atmospheric functions *ψ*_1_, *ψ*_2_, and *ψ*_3_ were computed from water vapor (*w*) measurements by the Moderate Resolution Imaging Spectrometer Atmospheric Profiles product (MOD07) and the following procedure proposed by Yu et al. (2014, equation 6):

(6) $$\left[\begin{array}{c} \psi_{1}\\ \psi_{2}\\ \psi_{3} \end{array}\right]=\left[\begin{array}{c} 0.0109\;\\ -0.0620\;\\ -0.0533\; \end{array}\begin{array}{c} \;0.0079\\ -0.4671\\ \;0.4013 \end{array}\begin{array}{c} \;0.0991\\ \;-1.2105\\ \;0.8585 \end{array}\;\begin{array}{c} \;1.0090\\ \;0.1176\\ -0.0443 \end{array}\right]\left[\begin{array}{c} \begin{array}{c} w^{3}\\ w^{2} \end{array}\\ \begin{array}{c} w^{1}\\ 1 \end{array} \end{array}\right]$$

*ε* was estimated following the procedures described in Sobrino et al. (2004), an NDVI‐based method for the estimation of *ε*. This study uses an NDVI threshold method (NDVI^THM^) for *ε* estimation, using calibration values presented for Landsat 8/TIRS band 10 (Skoković et al., 2014), as shown in equation (7).

(7) $$\varepsilon =\left\{\begin{array}{c} 0.979-0.046\rho_{\mathrm{red}}\\ 0.987P_{v}+0.971\times \left(1-P_{v}\right)\\ 0.99 \end{array}\right.\;\begin{array}{c} P_{v}=0\\ 0<P_{v}<1\\ P_{v}=1 \end{array}$$

where *ρ*_red_ is the reflectance at the red spectral band and *P*_*v*_ is the proportion of vegetation (also known as fractional vegetation cover). *P*_*v*_ can be calculated as presented in equation (8).

(8) $$P_{v}={\left(\frac{\text{NDVI}-{\text{NDVI}}_{s}}{{\text{NDVI}}_{v}-{\text{NDVI}}_{s}}\right)}^{2}$$

Where: NDVI_v_ is a NDVI value from a vegetation pixel and NDVI_s_ is a NDVI value from a soil pixel. In this study we used the values Sobrino and Raissouni (2000) proposed, 0.5 and 0.2, respectively, for NDVI_v_ and NDVI_s_.

### 2.5 Continuous Wavelet Transformation

Wavelet analysis involves transformation of a data series with a “mother” wavelet to produce “daughter” wavelets (Wiebe & Sturman, 2011). Thus, data are transformed into the frequency domain, which highlights the spectral characteristics of a time series. Johansson et al. (2009) presented an example of application of wavelet transformation for a dengue incidence time series and stated that the transformation was useful to differentiate multiannual patterns that made seasonal variation clear. In another study that applied wavelet analysis to dengue cases, Nagao and Koelle (2008) showed that there is a shift in the frequency dengue cases in Thailand. According to Johansson et al. (2009) there are two advantages of using wavelet technique for assessing the relationship between ENSO and dengue: (1) It allows the separation by time scale, and (2) it provides a way to measure nonstationary association. Thus, the wavelet technique can be considered as a cross correlation among a time signal and a set of wavelets.

In this study we used the continuous wavelet transformation to analyze how the frequency content of the weekly SSAT index data changes over time. The scale for this time series (*n* = 305) was set to 64. The computation of the wavelet and coefficients was performed using the Matlab wavelet toolbox (MathWorks, 2017). Compared with the Morlet wavelength, Wiebe and Sturman (2011), found the Haar wavelet to be more suitable for this type of analysis; hence, it was chosen for the temporal component of this study.

### 2.6 Temporal and Spatial Analysis

The above data sets were integrated to conduct the temporal and spatial assessments. For the temporal analysis we tried to identify a relationship between dengue cases and climatological variables. Since those are the variables with a high temporal variability, we complemented the relationship by applying the continuous wavelet transformation to the climatological data. To verify the benefit of this process, we applied the Pearson correlation and cross‐correlation analysis to the data with and without the continuous wavelet transformation. The temporal analysis provides information about the most vulnerable period for dengue outbreaks, helps identify coincidents events that could be their most important determinant variables, and can also help estimate the intensity of future dengue outbreaks. Parallely, remote sensing data such as LST and environmental indices and the spatial distribution of dengue cases per district were used for the spatial analysis. This analysis will provide a sense of where most of dengue cases are located as well as the environmental factors that correlate with high rates of dengue.

## 3 Results

### 3.1 Temporal Analysis

**Figure 3:**
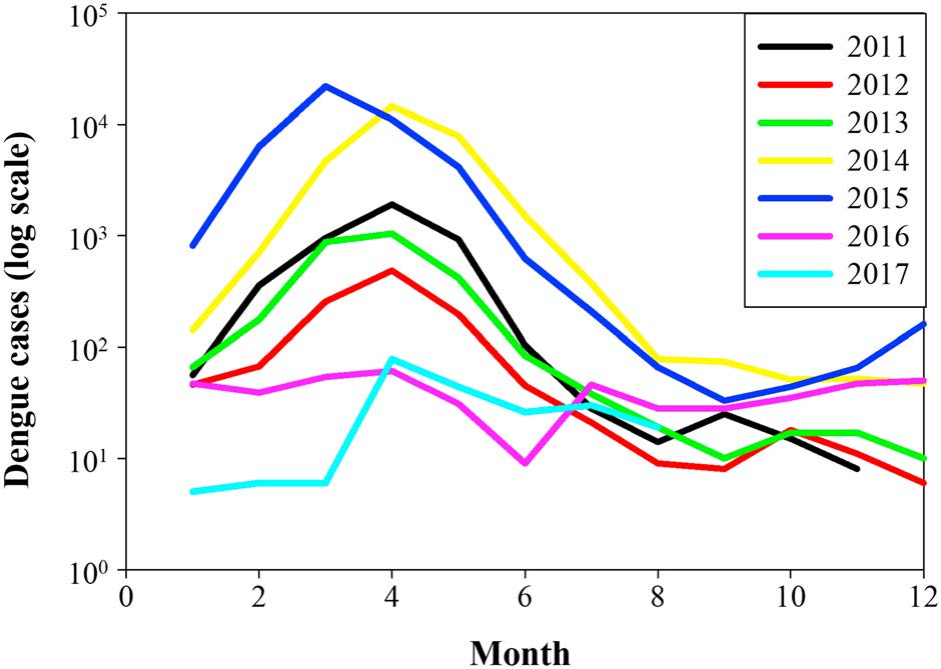
Distribution of confirmed dengue cases (in log scale) per epidemiological months in the municipality of São Paulo between the years of 2011 and 2017.

Figure 3 shows the monthly fluctuation of confirmed dengue cases along the studied years, with most of the dengue cases in São Paulo occurring at the end of the austral summer, during the first half of the year, when precipitation and temperature are the highest (as shown in Figure 2), but variable annual incidence range from as low as 475 cases in 2016 to 43,359 cases in the previous year (2015).

**Figure 4:**
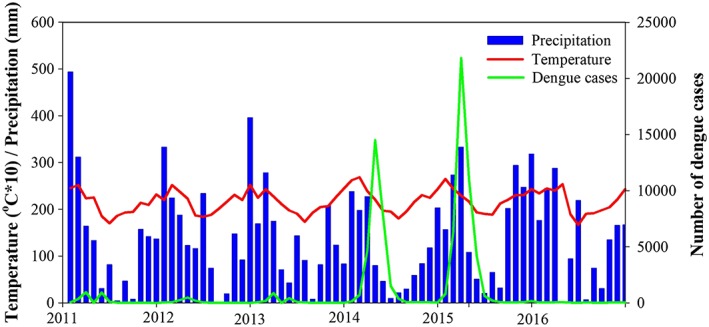
Time series of precipitation, temperature, and confirmed dengue cases (from 2011 to 2016).

Figure 4 presents the reported dengue cases with the climatic variables (precipation and temperature). No correlation was detected between the climatic variables (precipitation and temperature) and number of dengue cases. Correlation analysis with temperature resulted in a Spearman's rank coefficient (*rs*) value of 0.11 (*p* = 0.33) and a linear Pearson coefficient (*r*) value of 0.06 (*p* = 0.59). Similarly, a *rs* value of 0.09 (*p* = 0.40) and a *r* value of 0.10 (*p* = 0.36) were the correlation analysis found with precipitation.

**Figure 5:**
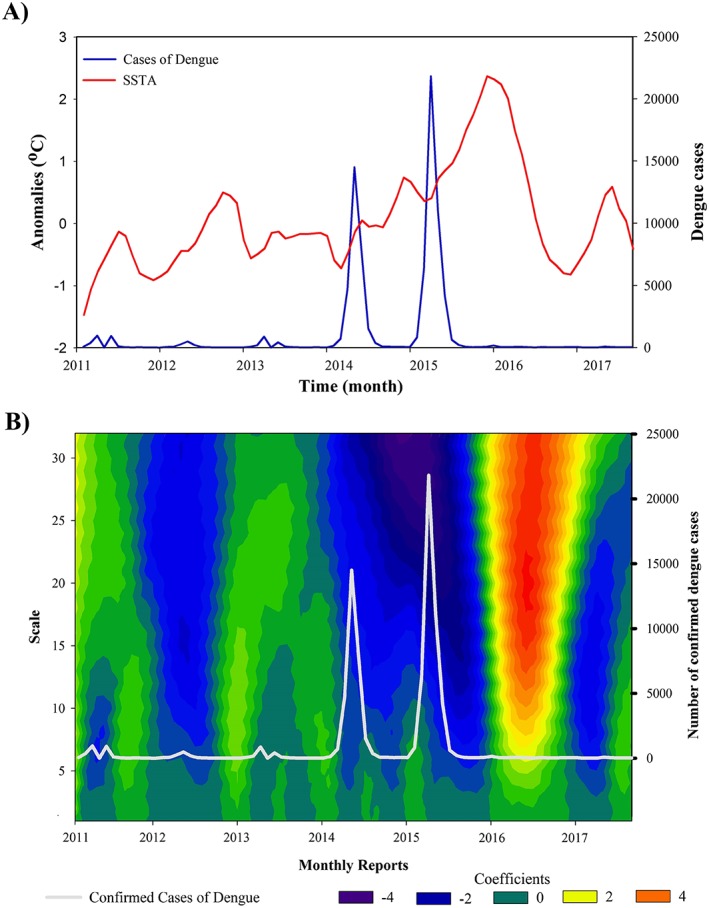
(a) Monthly SSTA index and dengue cases plot, and (b) wavelet transformation of the monthly SSTA index and dengue cases plot. SSTA = sea surface temperature anomalies.

Dengue cases by month in São Paulo city were plotted with the monthly SSTA index (Figure 5a). Figure 5a shows a pattern with peaks of dengue incidence within the first semester of each year (also observed on Figure 3). Increasing trends of SSTA coincided with dengue peaks in years 2011–2015, although a second and lower dengue peak occurred in 2013 that coincided with a SSTA peak. Conversely, after the highest SSTA peak of the study period (right at the beginning of 2016), there were almost imperceptible dengue peaks in years 2016 and 2017. To further explore the assessment of the relationship between weekly SSTA index and dengue cases, the Haar continuous wavelet transformation was applied to the weekly SSTA index time series and depicted in Figure 5b. The use of the Haar wavelet transformation (Figure 5b) suggests a relationship between coefficients (scalogram) and dengue cases (grey line). The scalogram for the weekly SSTA index shows increasing coefficient values tending to be blue with a tendency to green or red color for decreasing SSTA values. Decreasing coefficient values tend to happen at the second semester of the year right before the dengue season of the following year, which occurs at the first semester of the year (austral summer and austral autumn) during increasing coefficient values. However, 2016 and 2017 did not maintain this trend presumably in connection with the fact that the preceding year, 2015 (highest dengue peak of the study period) presented El Niño conditions during the entire year thus coinciding with a steady steep increase in SSTA (blue scalogram) and consequent drastic SSTA decrease (red scalogram) in the following year (2016). This year presented a drop in dengue incidence from 45,359 to 475 confirmed dengue cases, and the following year (2017) was even lower. The two years with the above normal peaks in dengue incidence (2014 and 2015) were predominantly El Niño years (NOAA, 2019) and presented an overall increasing trend for the monthly SSTA index with a predominant lower coefficient value for the Haar continuous wavelet transformation (<0, blue dominating color in the scalogram).

Our observations suggest that the continuous wavelet transformation of the SSTA index has a potential predictive ability over time that could complement the SSTA index. In terms of wavelet transformation the acute peaks in dengue cases appear to occur when coefficient values are 0 or negative (≤0, blue) but preceded by negative or slightly positive coefficient values (≤1, blue or olive green) the preceding year. Notice that a highly positive coefficient value (>2, light green or red) either during the normal dengue season (the first semester of the year) or at some point during the preceding year would result also in a low peak of dengue cases. Thus, lower wavelength transformation values tend to be associated with more dengue incidence. On the assumption that these described patterns for the SSTA curve and the coefficient values of the wavelength transformation would be a constant, it could be expected that 2017 would have few cases. This is because the last part of 2016 shows a strong decrease in the SSTA trend line and the wavelength transformation showed highly positive coefficient values (>2, light green or red). In fact, Figure 5 shows that 2017's dengue season did not present important peaks in confirmed dengue cases in São Paulo municipality. Only 214 cases were confirmed during the first eight months of 2017, while 2014 and 2015 had 30,066 and 45,359 cases, respectively, in the same corresponding period. The year 2016 differed with years 2011–2015 in the drastically higher coefficient value of wavelength transformation and lower monthly dengue incidence.

Other than our suggestion for a possible influence of ENSO measured through SSTA and wavelength transformation, we could not find a clear proposition to explain such a decrease. Although preventive measures sponsored by the public health authorities have been suggested as the cause of the decline in dengue incidence (Santiago, 2016), it is not clear why such supposedly measures did not protect against the huge Zika virus outbreak that occurred during the same year and whose etiologic agent is transmitted by the same vector, likewise why the peak in the Zika virus epidemic took place in 2016 despite the highly positive coefficient value in wavelet transformation. In fact, the decline in dengue incidence after 2015 could have also been influenced by the Zika virus epidemics (Fuller et al., 2017). The temporal coincidence between the two viruses might have cause some sort of competition that would have caused only one of the two to peak at a time. On the other hand, the reason why the Zika epidemic peaked in 2016 (and not in 2015 as in the dengue peak) might have been in part due to the timing of the Zika virus arrival into the naïve population and the time period it took to spread. For instance, we do not know if had the Zika virus arrived earlier its peak might had been even stronger due to even more favorable El Niño conditions and it might have coincided temporally with that of dengue or conversely, if one of the two would have just inhibited the other. In any case, the causes of this dengue decline are still not fully understood (Lopes et al., 2018).

**Figure 6:**
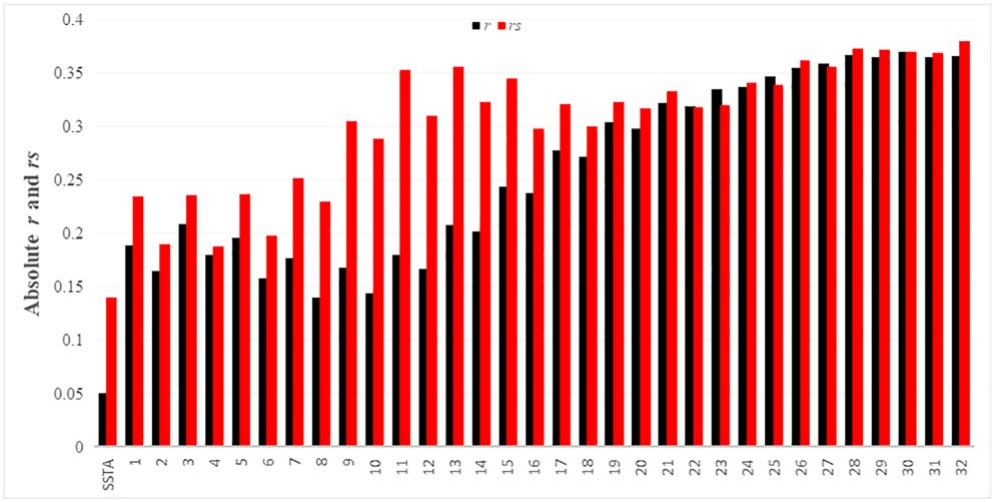
Absolute Pearson's correlation coefficient and absolute Spearman's rank correlation coefficient for the relationship between denge cases and both SSTA index and continuous wavelet transformation scales. SSTA = sea surface temperature anomalies.

We compared the continuous wavelet transformation scale values and SSTA index to the number of dengue cases via the Pearson correlation coefficient (*r*) and *rs*. Figure 6 shows the absolute *r* and *rs* values (black and red columns, respectively) between dengue cases and the SSTA index (first two columns) and between dengue cases and each scale from the continuous wavelet transformation (remaining columns). In both relationships the *rs* values were higher than the *r* values; however, for the continuos wavelet transformation scales this difference is minimal at higher scales. This indicates that, while SSTA was not linearly related to confirmed dengue cases, the transformed data were. Furthermore, the correlation between dengue cases and the SSTA was weaker (*rs* = 0.14) than that between dengue cases and the scale of the continuous wavelet transformation (*rs* = 0.38, at scale 32) when using a nonlinear relationship estimator. For a linear correlation, the transformated data was stronger (*r* = 0.37, at scale 30) than the SSTA (*r* = 0.05).

### 3.2 Spatial Analysis

#### 3.2.1 Surface Temperature of São Paulo

**Figure 7:**
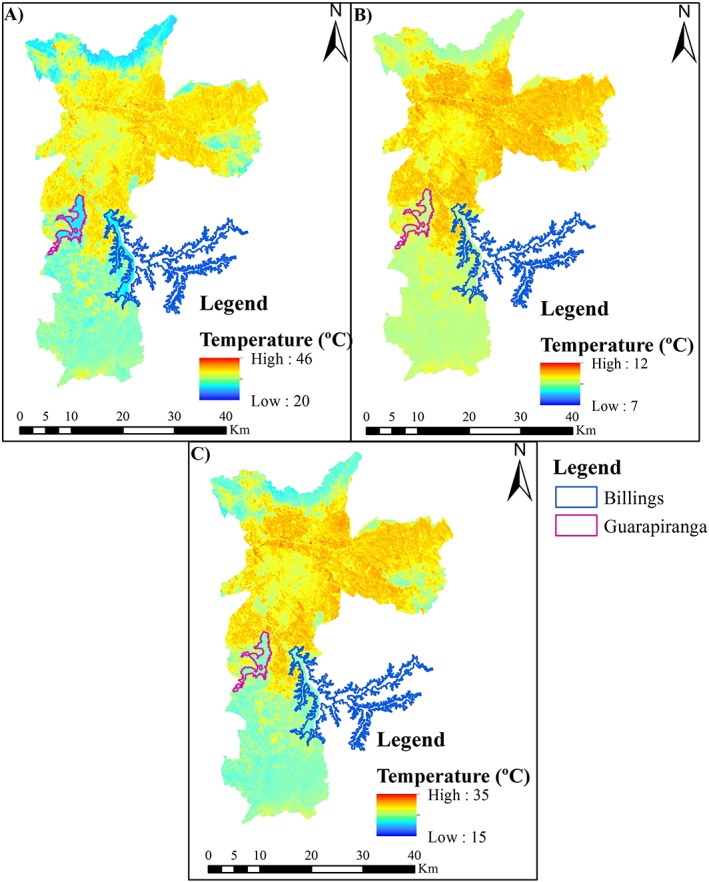
Land surface temperature for São Paulo city (a) for 2014, (b) for 2015, and (c) for 2016.

As expected, the urban area of São Paulo has a much higher LST than the suburbs; it is a major example of an urban heat island (UHI) in Brazil (Barros & Lombardo, 2016; Lombardo, 1985). Across dates (January 2015, February 2014, and April 2016) and different temperature scales, the spatial pattern in the images is the same, with higher temperatures in urban areas and lower temperatures in the suburbs (Figure 7). Barros and Lombardo (2016) recently discussed this spatial distribution of LST, as they identified different intensities of UHI occurring within São Paulo city. The authors also observed the presence of a park cool island that partially offsets the UHI effect in neighborhoods located in the midwest and south of the area that have more space with vegetation cover. Zipper et al. (2016) observed a similar effect in Madison, WI, USA.

#### 3.2.2 Spectral Indices of São Paulo City

Spectral indices (NDBI, NDVI, and NDWI) were computed for São Paulo municipality to explore the spatial distribution of dengue cases and remote estimated environmental indices. NDBI computation for São Paulo (Figure S1, see in the supporting information) showed a similar spatial pattern as LST results (Figure 7), with higher index values for urban areas and lower for the suburbs and water, respectively. This was expected since NDBI is an index to quantify the amount of building area, and it did not show a significant difference among 2014 (Figure S1a, see in the supporting information), 2015 (Figure S1b, see in the supporting information), and 2016 (Figure S1c, see in the supporting information). The Kruskal‐Wallis test for equal medians for these three data sets showed that there is no significant difference between medians (*H* = 4.363, *p* = 0.11). The NDVI showed a significant variation (Kruskal‐Wallis test, *H* = 6.445, *p* = 0.04) on the values computed over continental areas—not including lake areas (see Figure S2 in the supporting information). Although the NDVI calculated from 2015 (Figure S2b; see in the supporting information) and that from 2016 (Figure S2b, see in the supporting information) were similar, these were different to the NDVI calculated from 2014 (Figure S2a, see in the supporting information), especially over aquatic systems. These changes on NDVI values could be related to the 2014–2015 drought, which lowered the water level in all of São Paulo municipality reservoirs, causing problems such as eutrophication and algal blooms (which increases the vegetation signature within the water surface). The same could be observed for the results from the computation of the NDWI (Figure S3, see in the supporting information). In these results, for 2014 (Figure S3a, see in the supporting information) and 2015 (Figure S3b, see in the supporting information) the water bodies showed a medium value (close to the values observed for vegetation), while the NDWI values only increased in 2016 (Figure S3c, see in the supporting information). The spatial pattern of NDVI was the opposite of LST and NDBI, with lower values for water bodies and urban areas and higher values for the suburbs, while for the NDWI higher values were observed over water bodies and vegetation (in the suburbs) and lower values were observed in the urban area.

#### 3.2.3 Spatial Analysis

**Figure 8:**
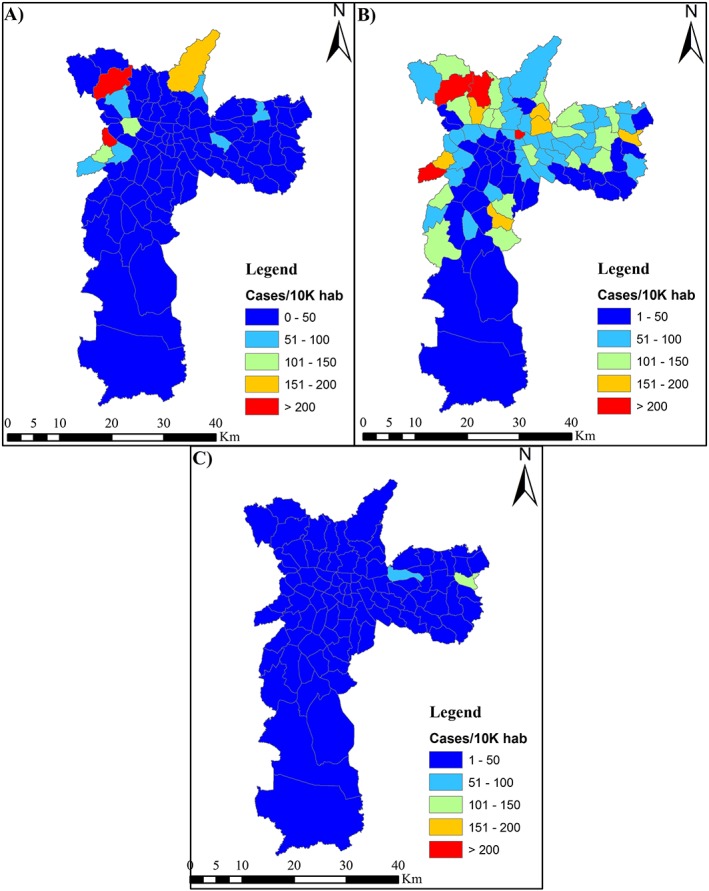
Reported dengue cases per 10,000 habitants per district of São Paulo city (a) for 2014, (b) for 2015, and (c) for 2016.

To evaluate the spatial characteristics of dengue outbreaks in São Paulo municipality, a data set of reported cases of dengue per district was used (São Paulo Secretariat of Health, 2017). Unfortunately, there is no data of confirmed cases at the district level; thus, reported cases were plotted per district per 10,000 habitants (Figure 8). The intensity observed in the temporal assessment of dengue cases in São Paulo was also observed in this spatial plot of reported cases, which showed more reported cases in 2015 (Figure 8b) and fewer in 2016 (Figure 8c). For 2014 (Figure 8a), the districts in the northern and northwestern parts of São Paulo municipality had the highest number of reported cases of dengue. This spatial pattern intensified in 2015 (Figure 8b). In 2016, however (Figure 8c), the eastern city districts had the highest number of reported cases.

Comparing the spatial distribution of reported dengue cases (Figure 8) to the mapping of LST (Figure 7) and that of NDBI (Figure S1, see in the supporting information) in São Paulo revealed that some of the districts with high dengue incidence had lower LST and NDBI than the main urban area. These spatial patterns could have been not expected since the primary vector (*A. aegypti*) is traditionally associated with warm temperatures, as does the dengue virus incubation (Messina et al., 2012), and with more urbanization, since urbanization is an important factor for the increase of endemicity of dengue (Gubler, 2004; Phillips, 2008; Russell et al., 2009). However, negative correlations with dengue incidence could occur with temperaures higher than the comfort zone of *A. aegypti* and conditions such as UHI could contribute to those higher temperatures and also explain negative correlations with urbanization. Such apparent discrepancy however, was not the case in all the districts with high dengue incidence as some others presented the expected coincidence of high dengue incidence with high LST and NDBI. Inconsistent patterns were also observed for the NDVI (see Figure S2 in the supporting information) and NDWI (Figure S3, see in the supporting information) as higher values were found for the northern and southern parts of São Paulo municipality. Yet the number of reported cases was higher just in few sections of the northern part of the municipality and lower in the southern part.

Correlation between average values of environmental indices per district and number of dengue cases per district were computed for the three years. The statistical analysis showed that dengue incidence was slightly correlated with LST (*r* = −0.25, *p* value < 0.001) but not with NDBI (*r* = −0.07, *p* value = 0.18), NDVI (*r* = −0.09, *p* value < 0.09) and NDWI (*r* = −0.075, *p* value < 0.20).

Based on these results presented, none of the selected spatial environmental factors seemed to clearly influence the spatial distribution of dengue cases. Therefore, to better evaluate the environmental variables, a forward stepwise regression was applied for the four remotely sensed estimated environmental variables (LST, NDBI, NDVI, and NDWI). For each variable, statistical estimators (minimum, maximum, average, and standard deviation) were calculated, using the variability within the area from each district, for a total of 16 variables. Table 2 summarizes the result of the forward stepwise regression, showing that minimum temperature is the environmental variable with the greatest *r* value possible (0.357) and the greatest determination coefficient (*R* ^2^) possible (0.127) with a standard error estimate of 77.45. Hence, minimum surface temperature was identified as an important variable for modeling the number of dengue cases. All other variables failed the *F* test and were not selected from the stepwise regression because of their high *p* values (Table S1, see in the supporting information).

**Table 2:** Summary of the Stepwise Regression for the 16 Environmental Variables Analyzed for Each District and Coefficients of the Model Showing Minimum Temperature as the Best Variable

Step #	Variables entered	Variables removed	r	*R* ^2^	*p* value	Coefficients
	Constant					115.619
1	Temperature minimum		0.357	0.127	<0.001	−3.254

## 4 Discussion

### 4.1 Temporal Assessment

The increase in the SSTA index between 2014 and 2015 affected São Paulo and caused the worst drought in the city since 1930 (Gutiérrez et al., 2014). During this period, the southeastern region of Brazil suffered from a 2‐year‐long drought, and São Paulo city was particularly affected. Escobar (2015) attributed this atmospheric anomaly to an atmospheric pachyderm which blocked the South Atlantic Convergence Zone, causing blockage of the southward flow of the water vapor from the Amazon Rain Forest from the northern region to the southeastern region of the country. Since the water vapor could not migrate to the southeastern region, precipitation levels in the peak of the summer were half the average in 2014 to 2015.

Concurrently, the number of confirmed dengue cases drastically increased in 2014 and 2015 (Figures 3, 4, 5) despite the lower than normal precipitation during this period. Yet, the normal intra‐annual increase in dengue incidence tends to coincide with that of precipitation, which in Sao Paulo occurs during the first semester of the year (Figures 2 and 3). A suggestion to explain similar inconsistency in the watershed of the Magdalena River in Colombia has been proposed by Eastin et al. (2014), Stanforth et al. (2016), and Ashby et al. (2017) who found dengue incidence to be importantly associated with temperature but not with precipitation in interanual trends. Interestingly, incidence is higher during the rainy months within the year, which are also the warmest, both in the Magdalena River watershed and in Sao Paulo (Figure 2). The authors suggest that temperature may be the important driver since human‐filled water containers make mosquito reproduction less dependent on precipitation, which is just associated without causation. Similar discussion has been proposed for other places (Moore et al., 1978; Pontes et al., 2000; Scott et al., 2000). In fact, Sao Paulo experienced slightly higher than normal temperature during the dengue outbreaks of 2014 and 2015. It is important to note that the end of 2014 and the entire 2015 presented El Niño conditions (NOAA, 2019).

In Colombia, higher incidence of dengue and malaria have been reported during El Niño years, which are characterized in this country by below normal precipitation and above normal temperature (Eastin et al., 2014; Gagnon et al., 2001; Kovats, 2000; Poveda et al., 2000). Similarly, higher incidences during drier years have been observed in Thailand (Ungchusak & Kunasol, 1988). Furthermore, Wu et al. (2018) did not find sufficient evidence to support a significant effect of precipitation on dengue fever. Beyond water storage, Cazales et al. (2005) observed that during drought periods, fast‐flowing rivers can recede into a series of stagnant pools, which are ideal for mosquito breeding. Thus, the positive increase in the SSTA index (Figure 5a) or the lower coefficient values (Figure 5b) appear to correlate with the higher number of dengue cases in São Paulo municipality, even during drought conditions. This same pattern was observed by Fan et al. (2014); however, they used the SOI to correlate with the dengue incidence in the Guangdong Province, China. Because of the difference in index as well as geographic location, the authors observed that negative values of SOI were related to warming caused by El Niño. These results suggest that climate variability will affect the potential for dengue epidemics, especially the increase of temperature and consequently drought conditions, which many regions experience in El Niño years.

It is important to highlight that the large dengue outbreaks of 2014 and 2015 occurred despite the implementation of the Brazilian National Plan for Dengue Control and the São Paulo Municipal Dengue Control Program, which started in 2002 and 2005, respectively (Taliberti & Zucchi, 2010). These programs were based on traditional interventions that rely on community visibility alone but not aided by temporal and spatial forecasting methods that could identify when and where control efforts should be made. The consideration of the SSTA index and the continuous wavelet transformation promises to be a helpful tool to observe the relationship between SSTA index (or El Niño effect) and reported dengue cases and thus be a potential pathway of research toward achieving an effective dengue incidence forcasting tool for public health management and policy makers.

These results align with other studies that assess the ENSO‐dengue relationships through other means and in other geographical areas, by identifying a lag between an El Niño event and a peak in dengue incidence (Eastin et al., 2014; Tipayamongkholgul et al., 2009). However, the effect of El Niño to the climate in São Paulo is not yet well understood. Grimm et al. (2000) did not find a significant effect of El Niño and La Niña cycles in the state of São Paulo. Contrarily, Rogers (1988) observed that in the vicinity of São Paulo the ENSO was responsible for the dry period between October and March. In addition, Aceituno (1988) observed that the Southern Oscillation and rainfall in the southeast of Brazil are negatively correlated, which corroborates Rogers' (1988) observations. Nevertheless, the opposite was also observed when El Niño years were related to the increase in rainfall in the period between November and January. All these mixed observations underline the lack of understanding of ENSO effects on the climate of São Paulo, thus highlighting the need for a better characterization of such effects in southeastern Brazil.

### 4.2 Spatial Assessment

Based on the visual interpretation of remotely estimated environmental variables, this study suggests that urban and rural districts have lower dengue incidences than suburban districts (located between urban areas and rural areas). In the suburban districts, a variance in the spatialized LST (Figure 7), NDBI (Figure S1), NDVI (Figure S2), and NDWI (Figure S3) was observed based on the standard deviation values from each district. Thus, the number of dengue occurrences could be related to the variability of environmental parameters. This could also help explain the spatial difference in the number of dengue cases between the southern and northern parts of the municipality. In the southern (lower standard deviation) part of São Paulo environmental factors varied less and the number of dengue cases was lower (Figure 8).

The overall low correlation between environmental variables and the number of reported dengue cases might be due to a complex interaction of counfonding effects of human hosts and their environment, which may have not been considered in this study. Although the annual temperature range in our study site roughly fluctuated between 12 and 32 °C (Figure 2), which includes the optimal survival for the vector, around 21 °C (Brady et al., 2013), it exceeded the lower limit for midrange temperatures of approximately 15–35 °C (Brady et al., 2013) and the optimal diurnal temperature range of 18–32 °C for mosquito survival and virus transmission (Eastin et al., 2014). Temperatures lower than 18 °C happened during midyear right after the dengue season, which strongly suggests temperature as a key factor determining the usual dengue season during the first half of the year.

The amount of daily hours with extreme air temperatures outside the optimal 18–32 °C range is being increasingly viewed as a potential environmental factor in recent ecological dengue studies (Eastin et al., 2014; Lambrechts et al., 2011). The 1,509 km^2^ of our study site could be enough to detect important spatial differences in the relationship between dengue incidence and diurnal temperature range among its 96 composing districts (between urban and suburban). Furthermore, the cited studies used air temperature, which exhibits lower daytime values and a much smaller diurnal range than the satellite‐derived LST used in this study. This adds to the uncertainty resulting from interchangeably using either one of these variables in urban areas despite their commonly well‐stablished relationship (Schwarz et al., 2012). Yet, a likely more important confounding factor interfering with the spatial assessment may be socioeconomic influences (Banu et al., 2011; Hayden et al., 2010). In fact, socioeconomic factors have been suggested to be important determinants in the incidence of diseases caused by viruses transmitted by *A. aegypti* (Moreno‐Madriñán & Turell, 2017; Reiter, 2001; Vasconcelos et al., 1998, 1999). A socioeconomic approach was previously used to derive a risk assessment for dengue in Cali, Colombia (Hagenlocher et al., 2013).

To explore the possibility that the poor relationship between dengue incidence and environmental variables might have been caused by socioeconomics, we conducted a complementary analysis where the socioeconomic classes were related to the number of reported dengue cases (Table 3). Dengue cases were distributed according to the reported residences's social‐economic class (percentage of families living within an *X* number of MWs) per district measured by the State System of Data Analysis (Fundação Sistema Estadual de Análise de Dados, SEADE, in Portuguese). Those districts with more families having more than 25 MWs have less dengue cases. This finding was expected since families with higher wages are more likely to live in wealthier districts, which may have better mitigation programs, sanitation, and living infrastructure. On the other hand, for families living on less than two MWs, the values of the *r* were close to 0 during the three analyzed years. This unexpected absence of relationship might be explained by confounding factors such as underreporting by people from the lowest socioeconomic class, where there is greater proportion of informal jobs, and thus less incentive to report sickness as compared to employees in the formal economy where workers need to present an excuse to their employers in order to get sick leave. Families living on 2–15 MWs (middle class), especially those in the middle range of 5–10 MWs, showed the most positive relationship with dengue incidence (*r* = >0.27, *p* < 0.01). Some differences to families living in richer neighborhoods may be based on the better vector control programs and living conditions of those neighborhoods, which reduce the opportunity of contact between humans and vector (Moreno‐Madriñán & Turell, 2017), and more common underreporting in poorer neighborhoods (Eastin et al., 2014).

**Table 3:** The Correlation Between Percentage of Residence in Each Economic Class (Based on MW) per District and Reported Dengue Cases

Year	Less than 2 MW	2 to 5 MW	5 to 10 MW	10 to 15 MW	15 to 25 MW	More than 25 MW
2014	−0.004 (*p* = 0.96)	0.075 (*p* = 0.46)	0.095 (*p* = 0.35)	0.000 (*p* = 0.99)	0.017 (*p* = 0.87)	−0.081 (*p* = 0.43)
2015	0.127 (*p* = 0.21)	0.238 (*p* < 0.02)	0.270 (*p* < 0.01)	0.038 (*p* = 0.71)	−0.149 (*p* = 0.14)	−0.251 (*p* < 0.02)
2016	−0.130 (*p* = 0.20)	0.170 (*p* = 0.10)	0.131 (*p* = 0.20)	0.015 (*p* = 0.88)	−0.131 (*p* = 0.20)	−0.165 (*p* = 0.10)

These results are in agreement with those by Vasconcelos et al. (1998, 1999). Both studies used a seroepidemiological analysis on the population of Fortaleza, Brazil, and Island of São Luiz, respectively. Vasconcelos et al. (1998) observed that people living with one to five MWs presented a higher dengue incidence as compared with people living with less than one MW or more than five MWs. Such study also provided an evaluation of educational levels and dengue contamination. It showed that people with middle and high school degrees were the most contaminated (45.2% and 49.6% of the population, respectively). On the other hand, people with no degree, with primary school degree, and with college degree have 39.6%, 34.3% and 38.9% of the population contaminated respectively. Although none of these studies provided an explanation for this apparently illogical relationship, our speculation of underreporting and informal employments in lower socioeconomic levels seems to be a plausible contributing factor. Therefore, understanding the social‐economic dynamics can be the starting point for future research to figure out the optimum population class for vectors control programs to target.

In general, it is understood that low socioeconomic‐related conditions favor the transmission of dengue and other mosquito‐borne diseases (Hagenlocher et al., 2013; Moreno‐Madriñán & Turell, 2017; Ramos et al., 2008; Reiter et al., 2003). Among the common influencing pathways for this relationship are the higher indoor temperatures due to the absence of air conditioning, more in‐and‐out access for mosquitoes due to open nonscreened windows, and sociocultural practices such as water storage as a result of the lack of continuous supply of piped water (Chareonviriyaphap et al., 2003; Eisen et al., 2014). In addition, lower socioeconomic neighborhoods have greater housing density, less access to vector control, and more likelihood of abandoned containers potentialy filled with water (Eastin et al., 2014). Among other possible risk facors are the less opportunity for early diagnosis and treatment, along with more likelihood for people to be outside as a result of less enhancements within households (Moreno‐Madriñán & Turell, 2018).

Although the spatial assessment did not provide an accurate spatial model, it could highlight some trends that should be investigated in the future, especially by policy makers in São Paulo municipality. Moreover, this same concluded observation could also be valid for other diseases which have the same vector, such as yellow fever, Zika (Wong et al., 2013), and chikungunya (CDC, 2016). It is important to emphasize not only that the results presented in this research are valid as a study case for São Paulo, Brazil, but that the same methodology and assessment could also apply to other tropical geographical localities.

## 5 Conclusions

In this work, we suggest the usefulness of considering the SSTA index trendlines and/or the continuous wavelet transformation with an SSTA time series to characterize the intensity and temporal distribution of dengue outbreaks in São Paulo municipality, Brazil. Although the effect of ENSO in São Paulo is not well understood and we do not imply a robust relationship with wavelet analysis for prediction, this technique could help estimate the intensity and magnitude of dengue incidences for the next season and be a starting point to develop a more advanced prediction tool. An interseasonal model using this technicque could be developed to initiate proactive public awareness, education campaigns, and planning of budgets and resources for prevention and mitigation. Thus, the use of this technique could be an important tool for policy makers as they budget for vector control programs. However, more validation studies in different geographic locations are needed for the standardization of this method. The spatial assessment of this study was not able to detect important influence from the remotely estimated environmental parameters considered except for minimum temperature for which just a low influence was detected (*r* = 0.357). Nevertheless, the MWs analysis implies that socioeconomic factors should also be considered to optimize a potential forecasting tool for locating areas at a higher risk for the occurrence of dengue outbreaks. The relevance of the presented study is the contribution to potential development of new strategies for the management of vector control programs. Although the coupling of temporal and spatial analysis was not conducted simultaneously in this study because of the low availability of cloud‐free images over São Paulo municipality, this could be a powerful management tool. The use of higher temporal resolution images could improve the frequency of spatial assessment, and it could be tied to the temporal analysis. Finally, although the present study focused on dengue outbreaks, these analyses may be adopted to investigate other diseases that share the same vector.

## Conflict of Interest

The authors declare no actual or perceived conflicts of interest.

## Supporting information

Supporting Information S1

## References

[gh2124-bib-0001] Aceituno, P. (1988). On the functioning of the Southern Oscillation in the South American sector. Part I: Surface climate. Monthly Weather Review, 116, 505–524.

[gh2124-bib-0004] Aguiar, M. , Rocha, F. , Pessanha, J. E. M. , Mateus, L. , & Stollenwerk1, M. (2015). Carnival or football, is there a real risk for acquiring dengue fever in Brazil during holidays seasons? Scientific Reports, 5, 8462 10.1038/srep08462 25684648 PMC4329556

[gh2124-bib-0005] Alvares, C. A. , Stape, J. L. , Sentelhas, P. C. , de Moraes‐Gonçalves, J. L. , & Sparovek, G. (2014). Köppen's climate classification map for Brazil. Meteorologische Zeitschrift, 22(6), 711–728. 10.1127/0941-2948/2013/0507

[gh2124-bib-0006] Ashby, J. , Moreno‐Madriñán, M. J. , Yiannoutsos, C. T. , & Stanforth, A. (2017). Niche modeling of dengue fever using remotely sensed environmental factors and boosted regression trees. Remote Sensing, 9, 328 10.3390/rs9040328

[gh2124-bib-0007] Banu, S. , Hu, W. B. , Hurst, C. , & Tong, S. L. (2011). Dengue transmission in the Asia‐Pacific region: Impact of climate change and socioenvironmental factors. Tropical Medicine & International Health, 16(5), 598–607. 10.1111/j.1365-3156.2011.02734.x 21320241

[gh2124-bib-0008] Barnston, A. G. , Chelliah, M. , & Goldenberg, S. B. (1997). Documentation of a highly ‐related SST region in the equatorial Pacific. Atmosphere‐Ocean, 35, 367–383. 10.1080/07055900.1997.9649597

[gh2124-bib-0009] Barros, H. R. , & Lombardo, M. A. (2016). A ilha de calor urbana e o uso e cobertura do solo em São Paulo‐SP. Geousp—Espaço e Tempo, 20(1), 160–177. 10.11606/issn.2179-0892.geousp.2016.97783

[gh2124-bib-0010] Bhatt, S. , Gething, P. W. , Brady, O. J. , Messina, J. P. , Farlow, A. W. , Moyes, C. L. , Drake, J. M. , Brownstein, J. S. , Hoen, A. G. , Sankoh, O. , Myers, M. F. , George, D. B. , Jaenisch, T. , Wint, G. R. W. , Simmons, C. P. , Scott, T. W. , Farrar, J. J. , & Hay, S. I. (2013). The global distribution and burden of dengue. Nature, 496, 504–507. 23563266 10.1038/nature12060 PMC3651993

[gh2124-bib-0011] Brady, O. J. , Johansson, M. A. , Guerra, C. A. , Bhatt, S. , Golding, N. , Pigott, D. M. , Delatte, H. , Grech, M. G. , Leisnham, P. T. , Maciel‐de‐Freitas, R. , Styer, L. M. , Smith, D. L. , Scott, T. W. , Gething, P. W. , & Hay, S. I. (2013). Modelling adult *Aedes aegypti* and *Aedes albopictus* survival at different temperatures in laboratory and field settings. Parasites & Vectors, 6(1), 351 10.1186/1756-3305-6-351 24330720 PMC3867219

[gh2124-bib-0012] Buczak, A. L. , Koshute, P. T. , Babin, S. M. , Feighner, B. H. , & Lewis, S. H. (2012). A data‐driven epidemiological prediction method for dengue outbreaks using local and remote sensing data. BMC Medical Informatics and Decision Making, 12(1), 124 10.1186/1472-6947-12-124 23126401 PMC3534444

[gh2124-bib-0014] Cazales, B. , Chavez, M. , McMichael, A. J. , & Hales, S. (2005). Nonstationary influence of El Nino on the synchronous dengue epidemics in Thailand. PLoS Medicine, 2, 313–318. 10.1371/journal.pmed.0020106 PMC1087219 15839751

[gh2124-bib-0015] Centers for Disease Control and Prevention (2010). Locally acquired dengue—Key West, Florida 2009–2010. MMWR, 59(19), 577–581. 20489680

[gh2124-bib-0016] Centers for Disease Control and Prevention . (2016). Surveillance and control of *Aedes aegypti* and *Aedes albopictus* in the United States. Retrieved from https://www.cdc.gov/chikungunya/pdfs/surveillance-and-control-of-aedes-aegypti-and-aedes-albopictus-us.pdf (Accessed October 24, 2017)

[gh2124-bib-0017] Chareonviriyaphap, T. , Akratanakul, P. , Nettanomsak, S. , & Huntamai, S. (2003). Larval habitats and distribution patterns of *Aedes aegypti* (Linnaeus) and *Aedes albopictus* (Skuse), in Thailand. Imsear, 34, 529–535. 15115122

[gh2124-bib-0018] Climate Prediction Center of the US National Weather Service (2017). Retrieved from: http://www.cpc.ncep.noaa.gov/data/indices/wksst8110.for

[gh2124-bib-0019] Eastin, M. D. , Delmelle, E. , Casas, I. , Wexler, J. , & Self, C. (2014). Intra‐and interseasonal autoregressive prediction of dengue outbreaks using local weather and regional climate for a tropical environment in Colombia. The American Journal of Tropical Medicine and Hygiene, 91(3), 598–610. 10.4269/ajtmh.13-0303 24957546 PMC4155567

[gh2124-bib-0020] Eisen, L. , Monaghan, A. J. , Lozano‐Fuentes, S. , Steinhoff, D. F. , Hayden, M. H. , & Bieringer, P. E. (2014). The impact of temperature on the bionomis of *Aedes* (Stegomyia) *aegypti*, with special reference to the cool graphic range margins. Journal of Medical Entomology, 51, 496–516. 10.1603/ME13214 24897844

[gh2124-bib-0021] Escobar, H. (2015). Drought triggers alarms in Brazil's biggest metropolis. Science, 347(6224), 812 10.1126/science.347.6224.812 25700493

[gh2124-bib-0022] Fan, J. , Lin, H. , Wang, C. , Bai, L. , Yang, S. , Chu, C. , Yang, W. , & Liu, Q. (2014). Identifying the high‐risk areas and associated meteorological factors of dengue transmission in Guangdong Province, China from 2005 to 2011. Epidemiology and Infection, 142, 634–643. 10.1017/S0950268813001519 23823182 PMC9161228

[gh2124-bib-0023] Focks, D. A. , Daniels, E. , Haile, D. G. , & Keesling, J. E. (1995). A simulation model of the epidemiology of urban dengue fever: Literature analysis, model development, preliminary validation, and samples of simulation results. American Journal of Tropical Medicine and Hygiene, 53(5), 489–506. 7485707 10.4269/ajtmh.1995.53.489

[gh2124-bib-0024] Fuller, D. O. , Troyo, A. , & Beier, J. C. (2009). El Niño Southern Oscillation and vegetation dynamics as predictors of dengue fever cases in Costa Rica. Environmental Research Letters, 4, 014011 10.1088/1748-9326/4/1/014011 PMC2745182 19763186

[gh2124-bib-0025] Fuller, T. L. , Calvet, G. , Genaro‐Estevam, C. , Rafael‐Angelo, J. , Abiodun, G. J. , Halai, U.‐A. , de Santis, B. , Sequeira, P. C. , Araujo, E. M. , Sampaio, S. A. , de Mendonça, M. C. L. , Fabri, A. , Ribeiro, R. M. , Harrigan, R. , Smith, T. B. , Gabaglia, C. R. , Brasil, P. , de Filippis, A. M. B. , & Nielsen‐Saines, K. (2017). Behavioral, climatic, and environmental risk factors for Zika and Chikungunya virus infections in Rio de Janeiro, Brazil, 2015‐16. PLoS ONE, 12(11), e0188002 10.1371/journal.pone.0188002 29145452 PMC5690671

[gh2124-bib-0026] Fundação Sistema Estadual de Análise de Dados (SEADE) . (2000). Distribuição dos Domicílios, por Faixa de Renda Familiar, segundo Distritos [Data File]. Retrieved from http://produtos.seade.gov.br/produtos/msp/ren/ren1_001.htm (Accessed October 24, 2017)

[gh2124-bib-0027] Gagnon, A. S. , Bush, A. B. G. , & Smoyer‐Tomic, K. E. (2001). Dengue epidemics and the El Niño Southern Oscillation. Climate Research, 19(1), 35–43. 10.3354/cr019035

[gh2124-bib-0028] Gao, B. C. (1996). NDWI—A normalized difference water index for remote sensing of vegetation liquid water from space. Remote Sensing of Environment, 58, 257–266. 10.1016/S0034-4257(96)00067-3

[gh2124-bib-0098] Grimm, A. M. , Barros, V. R. , & Doyle, M. E. (2000). Climate variability in southern South America associated with El Niño and La Niña events. Journal of climate, 13(1),35–58. 10.1175/1520-0442(2000)013<0035:CVISSA>2.0.CO;2

[gh2124-bib-0030] Gubler, D. J. (2004). The changing epidemiology of yellow fever and dengue, 1900 to 2003: Full circle? Comparative Immunology, Microbiology and Infectious Diseases, 27, 319–330. 10.1016/j.cimid.2004.03.013 15225982

[gh2124-bib-0031] Gutiérrez, A. P. A. , Engle, N. L. , Nys, E. D. , Molejón, C. , & Martins, E. S. (2014). Drought preparedness in Brazil. Weather and Climate Extremes, 3, 95–106. 10.1016/j.wace.2013.12.001

[gh2124-bib-0032] Gutman, G. G. (1991). Vegetation indices from AVHRR: An update and future prospects. Remote Sensing of Environment, 35, 121–136. 10.1016/0034-4257(91)90005-Q

[gh2124-bib-0033] Hadjimitsis, D. G. , & Clayton, C. (2009). Assessment of temporal variations of water quality in inland water bodies using atmospheric corrected satellite remotely sensed image data. Environmental Monitoring and Assessment, 159, 281–292. 10.1007/s10661-008-0629-3 19067211

[gh2124-bib-0034] Hagenlocher, M. , Delmelle, E. , Casas, I. , & Kienberger, S. (2013). Assessing socioeconomic vulnerability to dengue fever in Cali, Colombia: Statistical vs expert‐based modeling. International Journal of Health Geographics, 12, 36 10.1186/1476-072X-12-36 23945265 PMC3765508

[gh2124-bib-0035] Halsted, S. B. (2007). Dengue. The Lancet, 370, 1644–1652. 10.1016/S0140-6736(07)61687-0 17993365

[gh2124-bib-0036] Hayden, M. H. , Uejio, C. K. , Walker, K. , Ramberg, F. , Moreno, R. , Rosales, C. , Gameros, M. , Mearns, L. O. , Zielinski‐Gutierrez, E. , & Janes, C. R. (2010). Microclimate and human factors in the divergent ecology of *Aedes aegypti* along the Arizona, US/Sonora, MX border. EcoHealth, 7, 64–77. 20232228 10.1007/s10393-010-0288-z

[gh2124-bib-0037] Huang, B. , L'Heureux, M. , Lawrimore, J. , Liu, C. , Zhang, H. , Banzon, V. , Hu, Z.‐Z. , & Kumar, A. (2013). Why did large differences arise in the sea surface temperature datasets across the tropical Pacific during 2012? Journal of Atmospheric and Oceanic Technology, 30, 2944–2953. 10.1175/JTECH-D-13-00034.1

[gh2124-bib-0038] Instituto Brasileiro de Geografia e Estatística (IBGE) . (2012). Posição ocupada pelos 100 maiores municípios, em relação ao Produto Interno Bruto a preços correntes e participações percentuais relativa e acumulada, segundo os municípios e as respectivas Unidades da Federação—2012 [Data File]. Retrieved from ftp://ftp.ibge.gov.br/Pib_Municipios/2012/pdf/tab01.pdf (Accessed October 24, 2017)

[gh2124-bib-0039] Jimenez‐Munoz, J. C. , Cristobal, J. , Sobrino, J. A. , Soria, G. , Ninyerola, M. , & Pons, X. (2009). Revision of the single‐channel algorithm for land surface temperature retrieval from Landsat thermal‐infrared data. IEEE Transactions on Geoscience and Remote Sensing, 47, 339–349. 10.1109/TGRS.2008.2007125

[gh2124-bib-0040] Jimenez‐Munoz, J. C. , & Sobrino, J. A. (2003). A generalized single‐channel method for retrieving land surface temperature from remote sensing data. Journal of Geophysical Research, 108(D22), 4688 10.1029/2003JD003480

[gh2124-bib-0041] Jimenez‐Munoz, J. C. , Sobrino, J. A. , Skokovic, D. , Mattar, C. , & Cristobal, J. (2014). Land surface temperature retrieval methods from Landsat‐8 Thermal Infrared Sensor data. IEEE Geoscience and Remote Sensing Letters, 11(10), 1840–1843. 10.1109/LGRS.2014.2312032

[gh2124-bib-0042] Johansson, M. A. , Cummings, D. A. T. , & Glass, G. E. (2009). Multiyear climate variability and dengue—El Niño Southern Oscillation, weather, and dengue incidence in Puerto Rico, Mexico, and Thailand: A longitudinal data analysis. PLoS Medicine, 6(11), e1000168 10.1371/journal.pmed.1000168 19918363 PMC2771282

[gh2124-bib-0043] Kovats, R. S. (2000). El Niño and human health. Bulletin of the World Health Organization, 78, 1127–1135. 10.1590/S0042-96862000000900008 11019461 PMC2560836

[gh2124-bib-0044] Lambrechts, L. , Paaijmans, K. P. , Fansiri, T. , Carrington, L. B. , Kramer, L. D. , Thomas, M. B. , & Scott, T. W. (2011). Impact of daily temperature fluctuations on dengue virus transmission by *Aedes aegypti* . Proceedings of the National Academy of Sciences, 108(18), 7460–7465. 10.1073/pnas.1101377108 PMC3088608 21502510

[gh2124-bib-0045] Lemos, S. (2004). Minimum wage policy and employment effects: Evidence from Brazil. Economia, 5, 219–266. 10.1353/eco.2005.0007

[gh2124-bib-0047] Lombardo, M. A. (1985). Ilha de calor nas metrópoles: O exemplo de São Paulo. São Paulo. São Paulo, Brazil: Hucitec/Lalekla.

[gh2124-bib-0048] Lopes, T. R. R. , Silva, C. S. , Pastor, A. F. , & Silva Júnior, J. V. J. (2018). Dengue in Brazil in 2017: What happened? Revista do Instituto de Medicina Tropical de São Paulo, 60. 10.1590/S1678-9946201860043 PMC6103327 30133603

[gh2124-bib-0050] Luo, X. , & Li, W. (2014). Scale effect analysis of the relationships between urban heat island and impact factors: Case study in Chongqing. Journal of Applied Remote Sensing, 8(1), 084995 10.1117/1.JRS.8.084995

[gh2124-bib-0051] MathWorks . (2017). Wavelet toolbox (R2017b). Retrieved from: https://www.mathworks.com/help/wavelet/index.html (Accessed October 24, 2017)

[gh2124-bib-0052] McFeeters, S. K. (1996). The use of the Normalized Difference Water Index (NDWI) in the delineation of open water features. International Journal of Remote Sensing, 17(7), 1425–1432. 10.1080/01431169608948714

[gh2124-bib-0053] Messina, J. P. , Brady, O. J. , Pigott, D. M. , Golding, N. , Kraemer, M. U. G. , Scott, T. W. , Wint, G. R. W. , Smith, D. L. , & Hay, S. I. (2012). The many projected futures of dengue. Nature Reviews Microbiology, 13(4), 230–239. 10.1038/nrmicro3430 25730702

[gh2124-bib-0054] Moknatian, M. , Piasecki, M. , & Gonzalez, J. (2017). Development of geospatial and temporal characteristics for Hispaniola's Lake Azuei and Enriquillo using Landsat imagery. Remote Sensing, 9, 510 10.3390/rs9060510

[gh2124-bib-0055] Moore, C. G. , Cline, B. L. , Ruiz‐Tiben, E. , Lee, D. , Romney‐Joseph, H. , & Rivera‐Correa, E. (1978). *Aedes aegypti* in Puerto Rico: Environmental determinants of larval abundance and relation to dengue virus transmission. The American Journal of Tropical Medicine and Hygiene, 27, 1225–1231. 727328 10.4269/ajtmh.1978.27.1225

[gh2124-bib-0056] Moreno‐Madriñán, M. J. , Crosson, W. L. , Eisen, L. , Estes, S. M. , Estes, M. G. Jr. , Hayden, M. , Hemmings, S. N. , Irwin, D. E. , Lozano‐Fuentes, S. , Monaghan, A. J. , Quattrochi, D. , Welsh‐Rodriguez, C. M. , & Zielinski‐Gutierrez, E. (2014). Correlating remote sensing data with the abundance of pupae of the dengue virus mosquito vector, *Aedes aegypti*, in Central Mexico. ISPRS International Journal of Geo‐Information, 3, 732–749. 10.3390/ijgi3020732

[gh2124-bib-0057] Moreno‐Madriñán, M. J. , & Turell, M. (2018). History of mosquitoborne diseases in the United States and implications for new pathogens. Emerging Infectious Diseases, 24(5), 821 10.3201/eid2405.171609 29664379 PMC5938790

[gh2124-bib-0058] Moreno‐Madriñán, M. J. , & Turell, M. J. (2017). Factors of concern regarding Zika and other *Aedes aegypti*‐transmitted viruses in the U.S. Journal of Medical Entomology, 54(2), 251–257. 10.1093/jme/tjw212 28399294

[gh2124-bib-0059] Nagao, Y. , & Koelle, K. (2008). Decreases in dengue transmission may act to increase the incidence of dengue hemorrhagic fever. Proceedings of the National Academy of Sciences of the United States of America, 105, 2238–2243. 10.1073/pnas.0709029105 18250338 PMC2538904

[gh2124-bib-0060] National Institute of Meteorology (INMET in Portuguese) , (2017). via the Historical Research and Teaching Database. Retrieved from: http://www.inmet.gov.br/portal/index.php?r=bdmep/bdmep

[gh2124-bib-0061] National Oceanic and Atospheric Administration (NOOA) , (2019). Climate Prediction Center. Cold & warm episodes by season. Retrieved from: https://origin.cpc.ncep.noaa.gov/products/analysis_monitoring/ensostuff/ONI_v5.php

[gh2124-bib-0062] Patz, J. A. , Campbell‐Lendrum, D. , Holloway, T. , & Foley, J. A. (2005). Impact of regional climate change on human health. Nature, 438, 310–317. 10.1038/nature04188 16292302

[gh2124-bib-0063] Phillips, M. L. (2008). Dengue reborn: Widespread resurgence of a resilient vector. Environmental Health Perspectives, 116(09), A382–A388. 18795135 10.1289/ehp.116-a382 PMC2535648

[gh2124-bib-0064] Pinto, E. , Coelho, M. , Oliver, L. , & Massad, E. (2011). The influence of climate variables on dengue in Singapore. International Journal of EnvironmentalHealth Research, 21(6), 415–426. 10.1080/09603123.2011.572279 21557124

[gh2124-bib-0065] Pontes, R. J. S. , Freeman, J. , Oliveira‐Lima, J. W. , Hodgson, J. C. , & Spielman, A. (2000). Vector densities that potentiate dengue outbreaks in a Brazilian city. American Journal of Tropical Medicine and Hygiene, 62, 378–383. 11037781 10.4269/ajtmh.2000.62.378

[gh2124-bib-0066] Ponzoni, F. J. , & Shimabukuro, Y. E. (2009). Sensoriamento Remoto no Estudo da Vegetação, (1st ed.). São José dos Campos, Brazil: Parênteses.

[gh2124-bib-0067] Poveda, G. J. , Graham, N. E. , Epstein, P. R. , Rojas, W. , Velez, I. D. , Quiñones, M. L. , & Martens, P. (2000). Climate and ENSO variability associated to malaria and dengue fever in Colombia In Diaz H. F., & Markgraf F. (Eds.), El Niño and the Southern Oscillation, multiscale variability and global and regional impacts, (pp. 183–204). Cambridge: Cambridge University Press.

[gh2124-bib-0068] Prefeitura do Município de São Paulo . (2017). População Recenseada, Taxa de Crescimento Populacional e Densidade Demográfica [Data File]. Retrieved from http://infocidade.prefeitura.sp.gov.br/htmls/7_populacao_recenseadataxas_de_crescimento_1980_10745.html (Accessed October 24, 2017)

[gh2124-bib-0069] Ramos, M. M. , Mohammed, H. , Zielinski‐Gutierrez, E. , Hayden, M. H. , Lopez, J. L. R. , Fournier, M. , Muñoz, J. L. , Trujillo, A. R. , Hayden, M. H. , Banicki, A. A. , Lopez, J. L. R. , Ramos, M. M. , Morales, P. K. , Mohammed, H. , & Burton, R. (2008). Epidemic dengue and dengue hemorrhagic fever at the Texas–Mexico border: Results of a household‐based seroepidemiologic survey, December 2005. The American Journal of Tropical Medicine and Hygiene, 78(3), 364–369. 10.3410/f.724255595.793512904 18337327

[gh2124-bib-0070] Rasmussen, E. M. , & Carpenter, T. H. (1982). Variations in tropical sea surface temperature and surface wind fields associated with the Southern Oscillation/El Niño. Monthly Weather Review, 110, 354–384. 10.1175/1520-0493(1982)110<0354:VITSST>2.0.CO;2

[gh2124-bib-0071] Reiter, P. (2001). Climate change and mosquito‐borne disease. Environmental Health Perspectives, 109(Suppl 1), 141. 11250812 10.1289/ehp.01109s1141 PMC1240549

[gh2124-bib-0100] Reiter, P. , Lathrop, S. , Bunning, M. , Biggerstaff, B. , Singer, D. , Tiwari, T. , Baber, L. , Amador, M. , Thirion, J. , Hayes, J. , Seca, C. , Mendez, J. , Ramirez, B. , Robinson, J. , Rawlings, J. , Vorndam, V. , Waterman, S. , Gubler, D. , Clark, G. , & Hayes, E. (2003). Texas lifestyle limits transmission of dengue virus. Emerging Infectious Diseases, 9, 86–89. 10.3201/eid0901.020220 12533286 PMC2873752

[gh2124-bib-0072] Rogers, J. C. (1988). Precipitation variability over the Caribbean and tropical Americas associated with the Southern Oscillation. Journal of Climate, 1, 172–182.

[gh2124-bib-0073] Rouse, J. W. , Haas, R. H. , Schell, J. A. , & Deering, D. W. (1973). Monitoring vegetation systems in the Great Plains with ERTS In Freden S. C., Mercanti E. P., & Becker M. A. (Eds.), Proceedings of the Third ERTS Symposium NASA SP‐351, (Vol. 1, pp. 309–317). Washignton, DC, USA: NASA.

[gh2124-bib-0074] Russell, R. C. , Currie, B. J. , Lindsay, M. D. , Mackenzie, J. S. , Ritchie, S. A. , & Whelan, P. I. (2009). Dengue and climate change in Australia: Predictions for the future should incorporate knowledge from the past. Medical Journal of Australia, 190, 265–268. 19296793 10.5694/j.1326-5377.2009.tb02393.x

[gh2124-bib-0013] Santiago, T. (2016). Casos de dengue caem 76% no estado de São Paulo em 2016. Retrieved from: https://g1.globo.com/sao-paulo/noticia/alckmin-e-ministros-visitam-escolas-no-dia-de-combate-ao-mosquito-aedes-aegypti.ghtml

[gh2124-bib-0075] São Paulo Secretariat of Health . (2017). Dados epidemiológicos do município de São Paulo [Data File]. Retrieved from: http://www.prefeitura.sp.gov.br/cidade/secretarias/upload/casospordistrito.pdf (Accessed October 24, 2017)

[gh2124-bib-0076] São Paulo State Secretariat of Health . (2017). Dados Estatísticos [Data File]. Retrieved from: http://www.saude.sp.gov.br/cve-centro-de-vigilancia-epidemiologica-prof.-alexandre-vranjac/areas-de-vigilancia/doencas-de-transmissao-por-vetores-e-zoonoses/agravos/dengue/dados-estatisticos (Accessed October 24, 2017)

[gh2124-bib-0077] Schwarz, N. , Schlink, U. , Franck, U. , & Großmann, K. (2012). Relationship of land surface and air temperatures and its implications for quantifying urban heat island indicators—An application for the city of Leipzig (Germany). Ecological Indicators, 18, 693–704. 10.1016/j.ecolind.2012.01.001

[gh2124-bib-0078] Scott, T. W. , Morrison, A. C. , Lorenz, L. H. , Clark, G. G. , Strickman, D. , Kittayapong, P. , Zhou, H. , & Edman, J. D. (2000). Longitudinal studies of *Aedes aegypti* in Thailand and Puerto Rico: Population dynamics. Journal of Medical Entomology, 37, 77–88. 15218910 10.1603/0022-2585-37.1.77

[gh2124-bib-0079] Skoković, D. , Sobrino, J.A. , Jiménez‐Muñoz, J.C. , Sòria, G. , Julien, Y. , Mattar, C. , & Cristóbal, J. (2014). Calibration and Validation of land surface temperature for Landsat8‐TIRS sensor. In: *Land product validation and evolution*, ESA/ESRIN Frascati, Italy, 28‐30 January 2014. Retrieved from: https://earth.esa.int/documents/700255/2126408/ESA_Lpve_Sobrino_2014a.pdf (Accessed October 24, 2017)

[gh2124-bib-0080] Sobrino, J. A. , Jimenez‐Munoz, J. C. , & Paolini, L. (2004). Land surface temperature retrieval from Landsat TM 5. Remote Sensing of Environment, 90, 434–440. 10.1016/j.rse.2004.02.003

[gh2124-bib-0081] Sobrino, J. A. , & Raissouni, N. (2000). Toward remote sensing methods for land cover dynamic monitoring: Application to Morocco. International Journal of Remote Sensing, 21(2), 353–366. 10.1080/014311600210876

[gh2124-bib-0083] Stanforth, A. , Moreno‐Madriñán, M. , & Ashby, J. (2016). Exploratory analysis of dengue fever niche variables within the Río Magdalena Watershed. Remote Sensing, 8(9), 770 10.3390/rs8090770

[gh2124-bib-0084] Taliberti, H. , & Zucchi, P. (2010). Custos diretos do programa de prevenção e controle da dengue no Município de São Paulo em 2005. Revista Panamericana de Salud Pública, 27(3), 175–180. (in Portuguese) 20414506 10.1590/s1020-49892010000300004

[gh2124-bib-0085] Tipayamongkholgul, M. , Fang, C. T. , Klinchan, S. , Liu, C. M. , & King, C. C. (2009). Effects of the El Niño–Southern Oscillation on dengue epidemics in Thailand, 1996–2005. BMC Public Health, 9, 422 10.1186/1471-2458-9-422 19930557 PMC2785791

[gh2124-bib-0086] Ungchusak, K. , & Kunasol, P. (1988). Dengue haemorrhagic fever in Thailand 1987. Southeast Asian Journal of Tropical Medicine and Public Health, 19, 487–490. 3217826

[gh2124-bib-0087] USGS (2016). Landsat 8 (L8) data users handbook. Sioux Falls, South Dakota, USA: Department of the Interior U.S. Geological Survey.

[gh2124-bib-0088] Vasconcelos, P. F. C. , Lima, J. W. O. , Raposo, M. L. , Rodrigues, S. G. , Rosa, J. F. S. T. , Amorim, S. M. C. , Rosa, E. S. T. , Moura, C. M. P. , Fonseca, N. , & Rosa, A. P. A. T. (1999). Inquérito soro‐epidemiológico na Ilha de São Luis durante epidemia de dengue no Maranhão. Revista da Sociedade Brasileira de Medicina Tropical, 32(2), 171–179. 10.1590/S0037-86821999000200009 10228368

[gh2124-bib-0089] Vasconcelos, P. F. C. , Lima, J. W. O. , Rosa, A. P. A. T. , Timbó, M. J. , Rosa, E. S. T. , Lima, H. R. , Rodrigues, S. G. , & Rosa, J. F. S. T. (1998). Epidemia de dengue em Fortaleza, Ceará State, 1994: Inquérito soro epidemiológico aleatório. Revista de Saúde Pública, 32(5), 447–454. 10.1590/S0034-89101998000500007 10030061

[gh2124-bib-0090] Wiebe, A. , Sturman, A. , & McGowan, H. (2011). Wavelet analysis of atmospheric turbulence over a coral reef flat. Journal of Atmospheric and Oceanic Technology, 28(5), 698–708. 10.1175/2010JTECHA1485.1

[gh2124-bib-0091] Wong, P. S. , Li, M. I. , Chong, C. S. , Ng, L. C. , & Tan, C. H. (2013). *Aedes* (Stegomyia) *albopictus* (Skuse): A potential vector of Zika virus in Singapore. PLoS Neglected Tropical Diseases, 7, e2348 10.1371/journal.pntd.0002348 23936579 PMC3731215

[gh2124-bib-0092] Wu, X. , Lang, L. , Ma, W. , Song, T. , Kang, M. , He, J. , Zhang, Y. , Lu, L. , Lin, H. , & Ling, L. (2018). Non‐linear effects of mean temperature and relative humidity on dengue incidence in Guangzhou, China. The Science of the Total Environment, 628‐629, 766–771. 10.1016/j.scitotenv.2018.02.136 29454216

[gh2124-bib-0093] Xu, H. (2006). Modification of normalised difference water index (NDWI) to enhance open water features in remotely sensed imagery. International Journal of Remote Sensing, 27(14), 3025–3033. 10.1080/01431160600589179

[gh2124-bib-0094] Yu, X. , Guo, X. , & Wu, Z. (2014). Land surface temperature retrieval from Landsat 8 TIRS—Comparison between radiative transfer equation‐based method, split window algorithm and single channel method. Remote Sensing, 6, 9829–9852. 10.3390/rs6109829

[gh2124-bib-0095] Zha, Y. , Gao, J. , & Ni, S. (2003). Use of normalized difference built‐up index in automatically mapping urban areas from TM imagery. International Journal of Remote Sensing, 24, 583–594. 10.1080/01431160304987

[gh2124-bib-0096] Zipper, S. C. , Schatz, J. , Singh, A. , Kucharik, C. J. , Townsend, P. A. , & Loheide, S. P. II (2016). Urban heat island impacts on plant phenology: Intra‐urban variability and response to land cover. Environmental Research Letters, 11, 054023 10.1088/1748-9326/11/5/054023

