# Supplementary material for: Spatial‐Temporal Assessment of Environmental Factors Related to Dengue Outbreaks in São Paulo, Brazil
Source: Geohealth. 2019 Aug 21;3(8):202–17. doi: 10.1029/2019GH000186 (PMC7007072; doi:10.1029/2019GH000186)
Supplement: Supplementary file 1 — Supporting Information S1 [file GH2-3-202-s001.docx]

**Supplemental information for the manuscript:** Spatial-Temporal Assessment of Environmental Factors Related to Dengue Outbreaks in São Paulo, Brazil by Igor Ogashawara, Max-Jacobo Moreno Madriñán and Lin Li
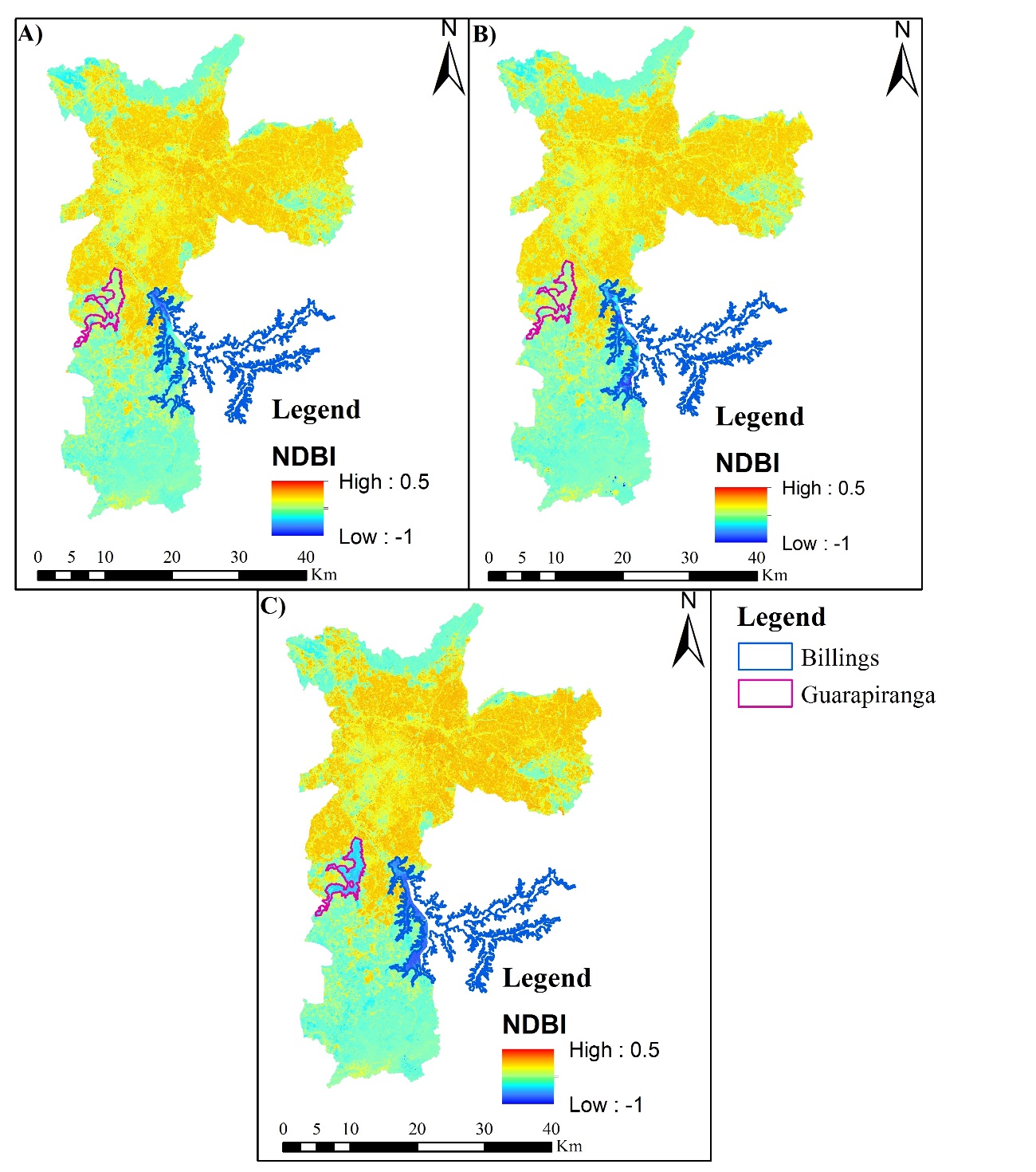


**Figure S1**. NDBI for São Paulo city A) for 2014, B) for 2015, C) for 2016.


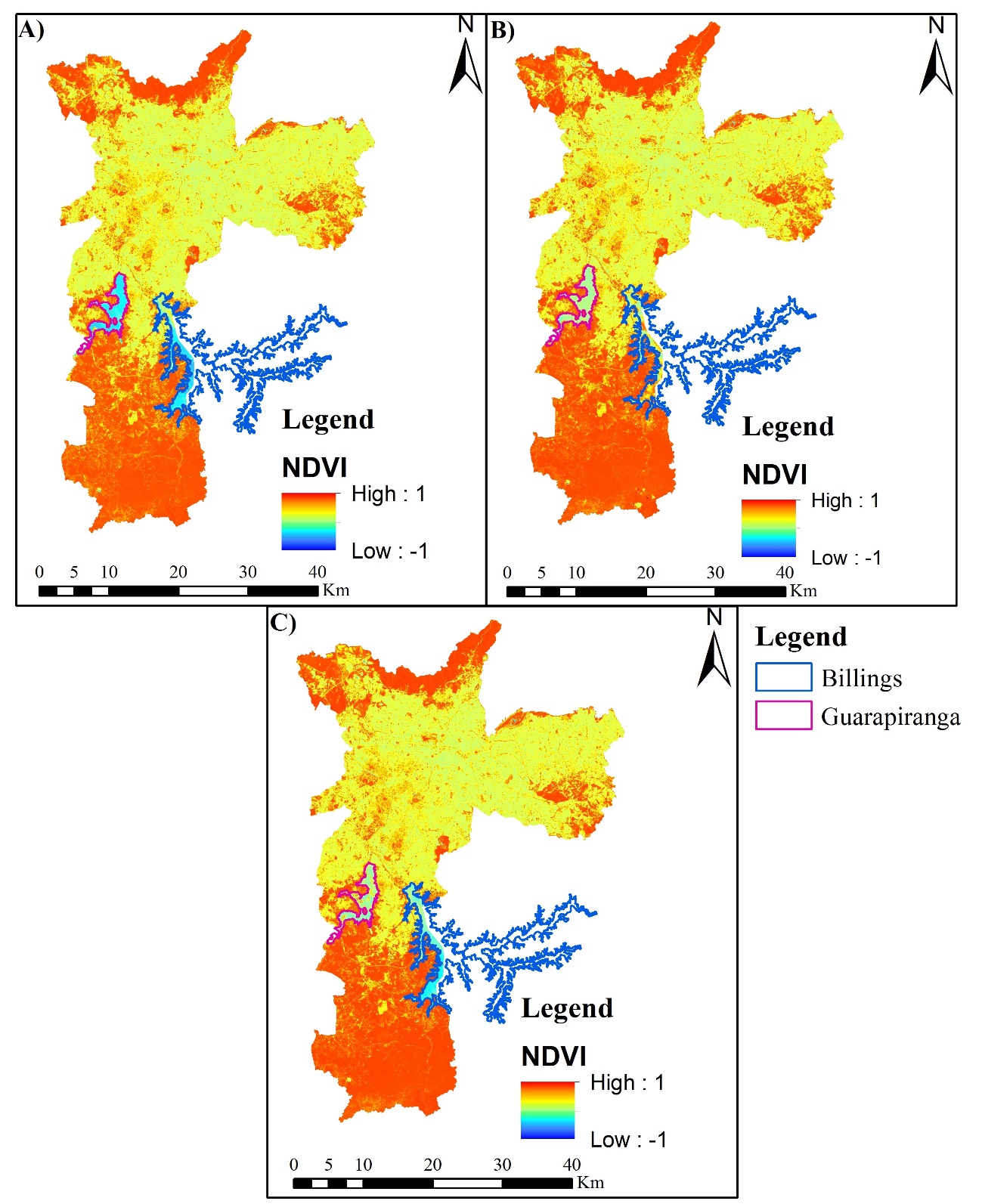


**Figure S2.** NDVI for São Paulo city A) for 2014, B) for 2015, C) for 2016.


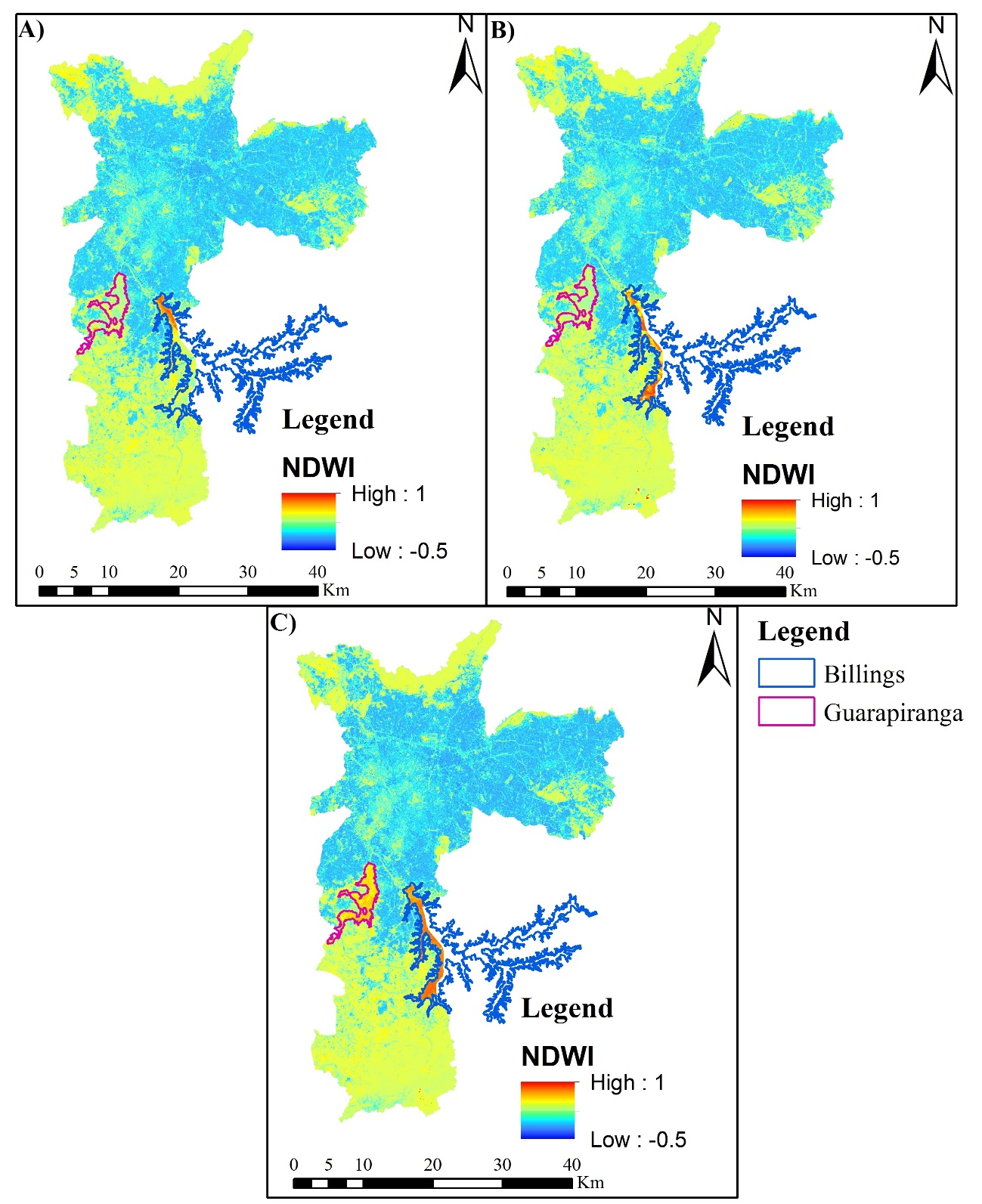


**Figure S3.** NDWI for São Paulo city A) for 2014, B) for 2015, C) for 2016.

**Table S1.** Variables not included in the forward stepwise regression.

| **Group** | **F-to-Enter** | **p-value** |
| --- | --- | --- |
| **Temp Max** | 0.0133 | 0.908 |
| **Temp Average** | 0.000116 | 0.991 |
| **Temp Std. Dev.** | 0.0079 | 0.929 |
| **NDBI Min.** | 0.737 | 0.391 |
| **NDBI Max.** | 0.00716 | 0.933 |
| **NDBI Average** | 0.0993 | 0.753 |
| **NDBI Std. Dev.** | 0.000269 | 0.987 |
| **NDVI Min.** | 0.0112 | 0.916 |
| **NDVI Max.** | 0.281 | 0.596 |
| **NDVI Average** | 0.542 | 0.462 |
| **NDVI Sdt. Dev** | 2.756 | 0.098 |
| **NDWI Min.** | 0.00705 | 0.933 |
| **NDWI Max.** | 0.735 | 0.392 |
| **NDWI Average** | 0.101 | 0.751 |
| **NDWI Std. Dev,** | 0.000211 | 0.988 |
